# Supplementary material for: ProteinShader: illustrative rendering of macromolecules
Source: BMC Struct Biol. 2009 Mar 30;9:19. doi: 10.1186/1472-6807-9-19 (PMC2672931; doi:10.1186/1472-6807-9-19)
Supplement: Additional file 1 — ProteinShader program without source code. This compressed file contains the complete ProteinShader program including associated libraries, but no source code. A README.txt file gives an overview of the ProteinShader distribution, and the index.html file in the help subdirectory has directions on getting started with the program as well as a set of tutorials. [file 1472-6807-9-19-S1.zip › ProteinShader-beta-0_9_4-binary/help/api/org/proteinshader/structure/class-use/Drawable.html]

Uses of Class org.proteinshader.structure.Drawable (ProteinShader API)


|  |  |  |
| --- | --- | --- |
| **Overview** \| **Package** \| **Class** \| **Use** \| **Tree** \| **Deprecated** \| **Index** \| **Help** | |  |
| PREV   NEXT | **FRAMES**    **NO FRAMES**     **All Classes** |  |


---


## **Uses of Class org.proteinshader.structure.Drawable**

| Packages that use Drawable | |
| --- | --- |
| **org.proteinshader.graphics.adapter** | Holds the StructureToGraphics class, which is used to manage the use of the drawing classes and OpenGL display lists. |
| **org.proteinshader.gui.viewing** | Holds important helper classes that the Renderer uses to control the view: Camera, Rotation, Lighting, *etc*. |
| **org.proteinshader.structure** | Holds the classes that store information from a Protein Data Bank file: Structure, Model, Chain, AminoAcid, Heterogen, Water, Atom, Bond, Helix, BetaStrand, Loop, *etc*. |
| **org.proteinshader.structure.sort** | Holds the DrawableSorter class, which is needed for sorting Drawable objects based on their distance from the camera. |
| **org.proteinshader.structure.visitor** | Holds Visitor classes that know how to traverse the hierarchy of objects held by class Structure. |
| **org.proteinshader.structure.visitor.modifiers** | Holds helper classes for the ModifierVisitor class, which is used to modify Drawable objects (Atoms, Bonds, and Segments). |

Uses of Drawable in org.proteinshader.graphics.adapter

| Methods in org.proteinshader.graphics.adapter with parameters of type Drawable | |
| --- | --- |
| `void` | `StructureToGraphics.draw(GL gl, Drawable d)`             Determines if a Drawable object is an Atom, Bond, or Segment, and then calls on the appropriate draw() method. |

Uses of Drawable in org.proteinshader.gui.viewing

| Methods in org.proteinshader.gui.viewing with parameters of type Drawable | |
| --- | --- |
| `double` | `Camera.calculateDepth(Drawable d)`             Calculates the depth (z-coordinate in camera space) of the xyz-center of the Drawable object. |
| `double` | `Camera.calculateDistance(Drawable d)`             Calculates the distance between the camera and the xyz-center of the Drawable object by using the Pythagorean theorem. |

Uses of Drawable in org.proteinshader.structure

| Subclasses of Drawable in org.proteinshader.structure | |
| --- | --- |
| `class` | `Atom`             Extends abstract class Drawable to add data that is unique to an atom (atomID, type, temperature, occupancy, radius, *etc.*). |
| `class` | `Bond`             Extends abstract class Drawable to add data that is unique to a bond between two atoms. |
| `class` | `Segment`             When a protein is drawn as a tube-like structure, a Segment is a length of the tube that corresponds to an AminoAcid. |

| Methods in org.proteinshader.structure that return Drawable | |
| --- | --- |
| `Drawable[]` | `Model.getOpaqueAtomsAndBonds()`             Copies the list of opaque Atoms and Bonds into an array and returns the array. |
| `Drawable[]` | `Model.getTranslucentDrawables()`             Copies the list of translucent Drawables into an array and returns the array. |

| Methods in org.proteinshader.structure with parameters of type Drawable | |
| --- | --- |
| `double` | `Drawable.distance(Drawable other)`             Returns the distance in angstroms between the xyz-center of the calling Drawable object and the xyz-center of the Drawable object given as an argument. |
| `Vec3d` | `Drawable.minus(Drawable other)`             Returns a vector created by subtracting the xyz-center of the Drawable given as an argument from the xyz-center of the calling Drawable object. |

Uses of Drawable in org.proteinshader.structure.sort

| Methods in org.proteinshader.structure.sort with parameters of type Drawable | |
| --- | --- |
| `void` | `DrawableSorter.ascendingMergeSort(Drawable[] d)`             Sorts an array of Drawable objects in ascending order based on camera distance. |

Uses of Drawable in org.proteinshader.structure.visitor

| Method parameters in org.proteinshader.structure.visitor with type arguments of type Drawable | |
| --- | --- |
| `void` | `VisibilityVisitor.setListsToFill(List<Segment> opaqueSegments, List<Drawable> opaqueAtomsAndBonds, List<Drawable> translucentList)`             Sets the lists to place opaque and translucent Drawable objects in. |
| `void` | `VisibilityVisitor.setListsToFill(List<Segment> opaqueSegments, List<Drawable> opaqueAtomsAndBonds, List<Drawable> translucentList)`             Sets the lists to place opaque and translucent Drawable objects in. |

Uses of Drawable in org.proteinshader.structure.visitor.modifiers

| Methods in org.proteinshader.structure.visitor.modifiers with parameters of type Drawable | |
| --- | --- |
| `void` | `DrawableModifier.modify(Drawable d)`             Modifies the visibility, RGBA color, and/or radius of the Drawable object given as an argument. |

---


|  |  |  |
| --- | --- | --- |
| **Overview** \| **Package** \| **Class** \| **Use** \| **Tree** \| **Deprecated** \| **Index** \| **Help** | |  |
| PREV   NEXT | **FRAMES**    **NO FRAMES**     **All Classes** |  |


---

# *Copyright © 2007-2008*
